# Dopamine signaling modulates microglial NLRP3 inflammasome activation: implications for Parkinson’s disease

**DOI:** 10.1186/s12974-022-02410-4

**Published:** 2022-02-16

**Authors:** Adrianne F. Pike, Francesca Longhena, Gaia Faustini, Jean-Marc van Eik, Iris Gombert, Maaike A. C. Herrebout, Mona M. H. E. Fayed, Michele Sandre, Tatiana Varanita, Charlotte E. Teunissen, Jeroen J. M. Hoozemans, Arianna Bellucci, Robert Veerhuis, Luigi Bubacco

**Affiliations:** 1 Department of Clinical Chemistry, Amsterdam Neuroscience, Neurochemistry Laboratory, Amsterdam UMC, Vrije Universiteit Amsterdam, De Boelelaan 1117, 1081 HV Amsterdam, The Netherlands; 2 Pharmacology Division, Department of Molecular and Translational Medicine, University of Brescia, Brescia, Italy; 3 Department of Biology, University of Padua, Padua, Italy; 4 Department of Pathology, Amsterdam Neuroscience, Neuropathology Laboratory, Amsterdam UMC, Vrije Universiteit Amsterdam, Amsterdam, The Netherlands; 5 Department of Psychiatry, Amsterdam Neuroscience, Amsterdam UMC, Vrije Universiteit Amsterdam, Amsterdam, The Netherlands

**Keywords:** Parkinson’s disease, NLRP3 inflammasome, Primary human microglia, Neuroinflammation, α-Synuclein, Dopamine, Potassium

## Abstract

**Background:**

Parkinson’s disease (PD) is characterized by the loss of nigral dopaminergic neurons leading to impaired striatal dopamine signaling, α-synuclein- (α-syn-) rich inclusions, and neuroinflammation. Degenerating neurons are surrounded by activated microglia with increased secretion of interleukin-1β (IL-1β), driven largely by the NLRP3 inflammasome. A critical role for microglial NLRP3 inflammasome activation in the progression of both dopaminergic neurodegeneration and α-syn pathology has been demonstrated in parkinsonism mouse models. Fibrillar α-syn activates this inflammasome in mouse and human macrophages, and we have shown previously that the same holds true for primary human microglia. Dopamine blocks microglial NLRP3 inflammasome activation in the MPTP model, but its effects in this framework, highly relevant to PD, remain unexplored in primary human microglia and in other in vivo parkinsonism models.

**Methods:**

Biochemical techniques including quantification of IL-1β secretion and confocal microscopy were employed to gain insight into dopamine signaling-mediated inhibition of the NLRP3 inflammasome mechanism in primary human microglia and the SYN120 transgenic mouse model. Dopamine and related metabolites were applied to human microglia together with various inflammasome activating stimuli. The involvement of the receptors through which these catecholamines were predicted to act were assessed with agonists in both species.

**Results:**

We show in primary human microglia that dopamine, l-DOPA, and high extracellular K^+^, but not norepinephrine and epinephrine, block canonical, non-canonical, and α-syn-mediated NLRP3 inflammasome-driven IL-1β secretion. This suggests that dopamine acts as an inflammasome inhibitor in human microglia. Accordingly, we provide evidence that dopamine exerts its inhibitory effect through dopamine receptor D1 and D2 (DRD1 and DRD2) signaling. We also show that aged mice transgenic for human C-terminally truncated (1–120) α-syn (SYN120 tg mice) display increased NLRP3 inflammasome activation in comparison to WT mice that is diminished upon DRD1 agonism.

**Conclusions:**

Dopamine inhibits canonical, non-canonical, and α-syn-mediated activation of the NLRP3 inflammasome in primary human microglia, as does high extracellular K^+^. We suggest that dopamine serves as an endogenous repressor of the K^+^ efflux-dependent microglial NLRP3 inflammasome activation that contributes to dopaminergic neurodegeneration in PD, and that this reciprocation may account for the specific vulnerability of these neurons to disease pathology.

## Background

Parkinson’s disease (PD) pathology manifests with several characteristic features. These include progressive neuronal degeneration that is prominent in the nigrostriatal dopaminergic system and results in the loss of basal ganglia dopamine (DA) inputs to elicit the onset of motor symptoms [1–4]. A second identifying aspect of PD pathology is the presence of intraneuronal and intraneuritic protein deposits known as Lewy bodies and Lewy neurites, respectively. These structures are enriched in α-synuclein (α-syn) fibrils [5–8]. Another characteristic contributor to PD pathology is neuroinflammation mediated by pro-inflammatory cytokines such as interleukin-1β (IL-1β) that are secreted mainly by activated microglia [9, 10]. Activation and proliferation of microglia in the SN, where the ratio of microglia to neurons is high even under basal conditions, is an important factor in the pathogenesis of PD as well as in vivo models thereof [11]. One pathological mechanism with established links to all of these fundamental facets of PD is the NLRP3 inflammasome [12].

The NLRP3 inflammasome is a key player in innate immunity and is the main driver of IL-1β secretion from microglia [13, 14]. It is a multimolecular scaffold whose main function is to sense, amplify, and broadcast proinflammatory signals from one cell to another by actuating cytokine secretion. The three traditionally accepted component proteins of the canonical NLRP3 inflammasome are: (1) the intracellular pattern recognition receptor NACHT domain-, leucine-rich repeat (LRR)-, and pyrin domain-containing protein 3 (NLRP3), which oligomerizes upon efflux of potassium (K^+^) from the cell to recruit (2) the small adaptor molecule known as apoptosis-associated speck-like protein containing a caspase activation and recruitment domain (CARD), or ASC, which further oligomerizes to recruit (3) the cysteine–aspartate protease-1 (caspase-1) [9, 14–17] Caspase-1’s recruitment to the inflammasome scaffold results in an increase of its local concentration, leading to its proximity-induced auto-activation, and caspase-1 in its active p33/p10 form then cleaves IL-1β for secretion [15, 18]. Since IL-1β is a potent pro-inflammatory cytokine, its production is regulated on multiple levels [19]. One means of regulating canonical NLRP3 inflammasome activation is the requirement for two separate signals to induce scaffold assembly and subsequent IL-1β processing by caspase-1 [20]; another is the intrinsic ability of caspase-1 to limit the extent of inflammasome activation through self-cleavage [15].

In contrast to canonical NLRP3 inflammasome activation, an alternative mode of inflammasome activation depending on caspases other than caspase-1 has been observed in human monocytes, non-human primate microglia, and mouse macrophages. Known as “non-canonical” inflammasome activation, it is induced by a single stimulus such as long-term (at least 16 h) treatment with LPS and involve caspases-4/-5 in humans and non-human primates, and in mice, the murine homologue caspase-11 [18, 21–26]. In non-canonical inflammasome activation, LPS serves as both priming and activation signal by binding initially to the TLR4 receptor at the cell surface and then, in the course of time, its uptake into the cell, where it activates the cytosolic caspases either directly or with the help of guanylate binding proteins. While LPS is a known activator of the non-canonical inflammasome, other activators such as oxidized lipids and NLRP1 have been proposed, and the still-unclear mechanisms regulating non-canonical inflammasome activation and resulting caspase-4 and -5 activity are a subject of ongoing study [18].

Mounting evidence suggests the involvement of the NLRP3 inflammasome mechanism in various aspects of PD pathology. NLRP3 and ASC protein levels are increased in the nigral microglia of PD patients in comparison to control patients and in the striatal microglia of mouse models of parkinsonism in comparison to WT mice [12]. α-Syn aggregates have been found to activate the NLRP3 inflammasome in mouse models to lead to IL-1β production in a delayed, caspase-1-dependent fashion [12, 27]. Activated caspase-1 has been shown to cleave α-syn at Asp121 to generate a C-terminal truncated form of the protein that is more prone to aggregation than full-length α-syn and serves as a further activation stimulus to the inflammasome [28]. We demonstrated previously the ability of α-synuclein to activate the NLRP3 inflammasome in primary human microglia leading to IL-1β processing in a manner that was dependent on NLRP3 oligomerization but unaffected by caspase-1 inhibition, suggesting the potential for non-canonical inflammasome activation involvement in this process in human microglia as opposed to mouse models [29].

Further evidence for a critical role for the NLRP3 inflammasome mechanism in PD is that pharmacological inhibition of its activation or knockdown of either NLRP3 or caspase-1 gene expression protected mice in various in vivo parkinsonism models against both dopaminergic neurodegeneration and the spreading of α-syn pathology [12, 30–32]. While this is a very important connection, the specific mechanistic influence of inflammasome activation on these pathological processes remains unknown.

Taken together, these observations implicate the NLRP3 inflammasome mechanism as a common link between each of the major characteristics of PD pathology: neuroinflammation, α-syn pathology, and dopaminergic neurodegeneration, warranting further investigation of the function of the inflammasome system in PD and its potential modulation. In this context, and given the concrete clinical significance of DA deficit in the manifestation of PD symptoms [33] and the successful application of the amino acid DA precursor levodopa (l-DOPA) to increase striatal DA levels as a gold standard therapeutic intervention to alleviate motor symptoms in PD patients [34, 35], it is salient that DA has been shown on a mechanistic level to inhibit various modes of NLRP3 inflammasome activation. This has been demonstrated in vitro in mouse macrophages and microglia as well as in vivo in the MPTP mouse model of parkinsonism, where DA exerts its inhibitory effect by regulating NLRP3 protein levels through DA receptor signaling [32].

It has been established that primary adult human microglia in culture express DA receptors DRD1, DRD2, DRD3, and DRD4, but not DRD5, and are highly chemotactically responsive to DA treatment [36]. However, the modulatory effects of DA have not yet been explored for the mechanisms of inflammasome activation in human microglia, nor for α-syn-mediated inflammasome activation in microglia of any species. To this end, we provide evidence from primary human and mouse microglia exposed to canonical and non-canonical stimuli and/or α-syn fibrils as well as from aged human C-terminally truncated (1–120) α-syn transgenic (SYN120 tg) mice, which exhibit α-syn aggregation and DA deficiency [37, 38], to suggest that DA receptor binding can indeed block PD-relevant NLRP3 inflammasome activation.

In the present study, the capacity of DA and its precursor l-DOPA to block canonical, non-canonical, and α-syn-mediated modes of NLRP3 inflammasome activation in primary human microglia was examined and characterized, as this inhibition could have implications for the etiology of PD. Downstream metabolites of DA were tested as well, specifically the neurotransmitter and paracrine hormone norepinephrine and its derivative epinephrine, given that norepinephrine in particular has a neuroprotective effect via β-adrenoceptor signaling [39, 40]. We identified receptors involved in DA-mediated inhibition using agonists for DRD1 and DRD2: SKF 82958, SKF 38393, and LY 171555 (also known as quinpirole).

Elevated extracellular K^+^ is a well-known inhibitor of canonical NLRP3 inflammasome activation in mouse macrophages and human monocytes, as charge and concentration gradient-regulated K^+^ efflux from the cell is usually, but not always, a signal converging from structurally diverse stimuli to trigger NLRP3 oligomerization leading to inflammasome scaffold assembly and IL-1β processing [16, 41–46]. Voltage-gated Kv1 potassium channels have been shown to be responsible for DA release in the brain in vivo [47], tying K^+^ flux to DA release, so the ability of elevated extracellular K^+^ to block different modes of NLRP3 inflammasome activation in human microglia was considered relevant to PD and was, therefore, also explored.

## Methods

### Isolation and culture of primary microglia from human brain tissue

Adult primary human microglia were isolated from post-mortem brain specimens by density gradient centrifugation essentially as previously described by [48]. Corpus callosum or subventricular cortical white matter tissue specimens were acquired from rapid autopsy according to the standard protocols of the Netherlands Brain Bank [49], with informed donor consent having been obtained from either patients or next of kin during life. Permission for the use of human brain tissue for in vitro research in compliance with the Declaration of Helsinki and approval was granted by the Medical Ethics Committee at VUmc. All tissue was collected in Dulbecco’s modified Eagle medium (DMEM, Gibco) supplemented with 0.1% gentamycin (Gibco). The isolated microglia were cultured in medium comprising 1:1 DMEM and Ham F10 (Gibco) supplemented with 10% v/v heat inactivated fetal bovine serum (Hyclone, Thermo Fisher Scientific), a mixture of 100 IU/mL penicillin and 50 µg/mL streptomycin (Gibco), and 0.5% l-glutamine (Gibco). For experimentation, microglia were seeded in 24- or 48-well uncoated culture plates (Corning Costar) and incubated at 37° with 5% CO_2_. 24 h after isolation, the microglia were treated with 25 ng/mL granulocyte macrophage colony stimulating factor (recombinant human GM-CSF, Immunotools) to allow for better adherence and proliferation, after which the medium was replaced with fresh culture medium approximately every 72 h. Microglia were utilized in experiments between days 6 and 10 post-isolation, and the results of each experiment as indicated in figures represent an individual microglial culture from an individual patient.

### Cell culture and reagents

The human monocyte-like leukemia cell line THP-1 was obtained from ATCC (Rockville, MD, CLS Cat# 300356/p804_THP-1, RRID:CVCL_0006). The cells were cultured in Roswell Park Memorial Institute (RPMI) 1640 medium with GlutaMAX (Gibco) supplemented with 10% heat inactivated FBS (Hyclone, Thermo Fisher Scientific, Breda, the Netherlands) and a mixture of 100 IU/mL penicillin and 50 µg/mL streptomycin (Gibco). The THP-1 cells were differentiated by the addition to culture medium upon seeding in 24- or 48-well uncoated culture plates (Corning Costar, Amsterdam, the Netherlands) of 100 ng/mL phorbol 12-myristate 13-acetate (PMA, Sigma-Aldrich, cat.# P-8139, Mechelen, Belgium) for 72 h prior to exposure. Lipopolysaccharide (LPS) from *E. coli* O55:B5 was from Sigma-Aldrich (catalog no. L-2880). Nigericin was from Adipogen (cat.# AG-CN2-0020-M005). Recombinant human α-syn was overexpressed and purified in monomeric form from *E. coli* and then aggregated into fibrils (250 mM nominal concentration of monomers to form 3615 µg/mL aqueous fibril stock solution) as previously reported [50]. No endotoxin contamination was detectable in the α-Syn monomer and fibril preparations, as determined with a limulus amoebocyte lysate (LAL) assay. Fibrils were not sonicated prior to application in culture. Note: nominal concentrations of α-syn fibrils as reported refer to the concentrations of the monomer solutions from which they were fibrillized. l-DOPA (cat.# 3788) and SKF82958 hydrobromide (cat.# 5719) were from Tocris Biosciences. Dopamine (cat.# H8502-5G), l-norepinephrine hydrochloride (cat.# 74480), (-)-epinephrine (cat.# E4250), isoprenaline hydrochloride (cat.# I5627), and (-)-quinpirole hydrochloride (LY171555, cat.#Q102) were all from Sigma Aldrich. KCl salt (for analysis, Emsure, cat.# 104936) was from Merck Millipore.

### Exposure conditions

PMA-differentiated (and thus primed) THP-1 cells in 24- (450,000 cells/well) culture plates were washed once with PBS to remove serum proteins left over from culture medium. Cells were exposed to LPS (50 ng/mL) as a time-matched, non-canonical NLRP3 inflammasome activation control stimulus or to various concentrations of α-syn fibrils at 5 µM (nominal concentration) in 250 µL serum-free exposure medium for 18–24 h. For canonical activation, PMA-differentiated THP-1 cells were exposed to 10 µM nigericin in 250 µL serum-free exposure medium for 30 min (additional LPS priming was not required for this cell type). GM-CSF-treated primary human microglia in 24- or 48-well plates after 6–10 days in culture were also washed once with PBS and were exposed to 20 ng/mL LPS or 5 µM α-syn fibrils in 250 µL serum-free exposure medium for 18–24 h. For canonical activation, primary human microglia were exposed to 20 ng/mL LPS for 3.5 h in 250 µL serum-free exposure medium followed by the addition of 5 µL nigericin solution to a final concentration of 10 µM for 30 min. In inhibition experiments, 125–1 mM DA, 1 mM l-DOPA, 10–50 µM isoprenaline, norepinephrine, or epinephrine, or 100 µM SKF82958 or quinpirole were administered at the time of addition of the initial stimulus (i.e., for canonical activation experiments, upon LPS priming) and left in the system for the duration of the exposure. Both DA and l-DOPA were prepared, stored, and handled as much in the dark as possible to avoid light-induced oxidation. After cell exposure, supernatants were collected immediately and centrifuged at 0.8×*g* for 5 min at room temperature to remove any cell debris. Cell lysates were collected by scraping with a pipet tip in lysis buffer [0.5% NP-40 in PBS with protease inhibitor cocktail (cOmplete mini, EDTA-free, Roche, Woerden, the Netherlands)] on ice, and cell debris was removed via centrifugation at 21,000×*g* for 10 min at 4 °C. Cleared supernatants and cell lysates were stored at − 20 °C until testing.

### ELISA analysis

The concentration of IL-1β in culture supernatants was determined with a sandwich enzyme-linked immunosorbent assay (ELISA) specific for the detection of human IL-1β (PeliKine compact kit, Sanquin, Amsterdam, the Netherlands).

### Viability assays

The lactate dehydrogenase leakage assay (LDH, Pierce, Breda, the Netherlands), to assess membrane integrity, and the methyl tetrazolium assay (MTT, Sigma M-2128, Mechelen, Belgium), to assess mitochondrial function, were performed according to manufacturer’s protocols. For the MTT assay, the supernatants were first removed from the cell culture and 0.25 mg/mL MTT in complete cell culture medium was added. After 2 h at 37 °C, the MTT solution was removed and 100 µL DMSO was added to each well. The plate was shaken for 1 min and absorbance was measured at a wavelength of 540 nm.

### Animals

C57BL/6J wt mice (C57BL/6J) (Charles River, Wilmington, MA), and SYN120 tg male mice that express C-terminally truncated form of α-syn of 120 aa (SYN120) under the control of rat tyrosine hydroxylase promoter on a mouse C57BL/6JOlaHsd α-syn null background [37] were used in this study at 12 months of age. Concerning the pharmacological treatments, the DRD1 agonist SKF38393 (Sigma, St Louis, MO, USA) was diluted in a sterile solution of NaCl (0.9 mg/mL) and injected intraperitoneally (i.p.) in 11-month-old SYN120 tg mice at a dose of 10 mg/kg every day for 1 month [51]. Mice treated with vehicle were injected with NaCl (0.9 mg/mL). Animals were maintained under a 12 h light–dark cycle at a room temperature (rt) of 22 °C and had ad libitum food and water. All experiments were made in accordance to Directive 2010/63/EU of the European Parliament and of the Council of 22 September 2010 on the protection of animals used. All experimental procedures conformed to the National Research Guide for the Care and Use of Laboratory Animals and were approved by the Animal Research Committees of the University of Brescia (Protocol Permit 04/10 and 719/2015-PR). All efforts were made to minimize animal suffering and to reduce the number of animals used.

### Double immunofluorescence staining

Twelve-month-old C57BL/6J and Tg120 mice were anesthetized with chloral hydrate 400 mg/kg i.p. (Sigma-Aldrich, St. Louis, MO, USA) and transcardially perfused with ice-cold Immunofix (4% PFA, Bio-optica, Milan). Brains were post-fixed for 4 h in Immunofix and conserved in 18% sucrose (Sigma-Aldrich, St. Louis, MO, USA) in PBS 0.1 M. The brains were then cut into 25 μm coronal sections with a cryostat and conserved in 60% glycerol. After permeabilization in PBS 0.1 M supplemented with 20% methanol and 0.3% Triton X-100, free floating striatum slices were incubated for 1 h at rt in blocking solution (3% Normal Goat Serum (NGS, Thermo Fisher, Waltham, MA, USA), 2% Bovine Serum Albumin (BSA, Sigma-Aldrich, St. Louis, MO, USA), 0.3% Triton-X100 in PBS 0.1 M) and then with the primary antibody (NLRP3 1:200, Adipogen, San Diego, CA, USA) in blocking solution overnight at 4 °C. The following day, slices were washed with 0.3% Triton-X100 PBS 0.1 M and incubated with the biotin-conjugated secondary antibody (VectorLabs, Burlingame, CA, USA) in 0.3% Triton-X100 PBS 0.1 M plus 1 mg/mL BSA for 1 h at rt and then with AlexaFluor594™-conjugated streptavidin (Thermo Fisher, Waltham, MA, USA) in water for 1 h at rt. After three washes in 0.3% Triton X-100 PBS, slices were incubated for 2 h at rt with the second primary antibody (ASC 1:500 Adipogen, San Diego, CA, USA or Ionized calcium binding adaptor molecule 1 (Iba1) 1:500, Wako, Osaka, Japan) prepared in blocking solution, followed by incubation for 1 h at rt with the optimal fluorochrome-conjugated secondary antibody (VectorLabs, Burlingame, CA, USA). Finally, cell nuclei were counterstained with Hoechst (Sigma-Aldrich, St. Louis, MO, USA), and the slices were first incubated with TrueBlack (VectorLabs, Burlingame, CA, USA) to minimize auto-fluorescence and mounted onto Superfrost slides using Vectashield mounting medium for fluorescence (VectorLabs, Burlingame, CA, USA).

### Confocal microscopy

Slides were observed by a LSM 880 Zeiss confocal laser microscope (Carl Zeiss S.p.A., Milan, Italy) with the following laser sets: *λ* = 405–488–543. The height of sections scanning was 1 μm. Eight images per striatum (512 × 512 pixels) were then reconstructed using ZEISS ZEN Imaging Software (Carl Zeiss S.p.A., Milan, Italy) and analyzed with ImageJ (NIH, Bethesda, MA, USA).

### Statistical analysis

Two-way ANOVA with Bonferroni’s multiple testing correction or nonlinear regression was used to analyze ELISA data from THP-1 and human and mouse microglia experiments. One-way ANOVA with Bonferroni’s multiple testing correction was used to analyze immunofluorescence quantification differences between SKF38393-treated SYN120 tg, vehicle treated SYN120 tg, SYN120 tg and WT mice. All statistical analyses were performed using GraphPad Prism v.7 or 8 (GraphPad Software, San Diego, CA, USA).

## Results

### Dopamine inhibits canonical, non-canonical, and α-syn-mediated NLRP3 inflammasome activation in primary human microglia

**Fig. 1:**
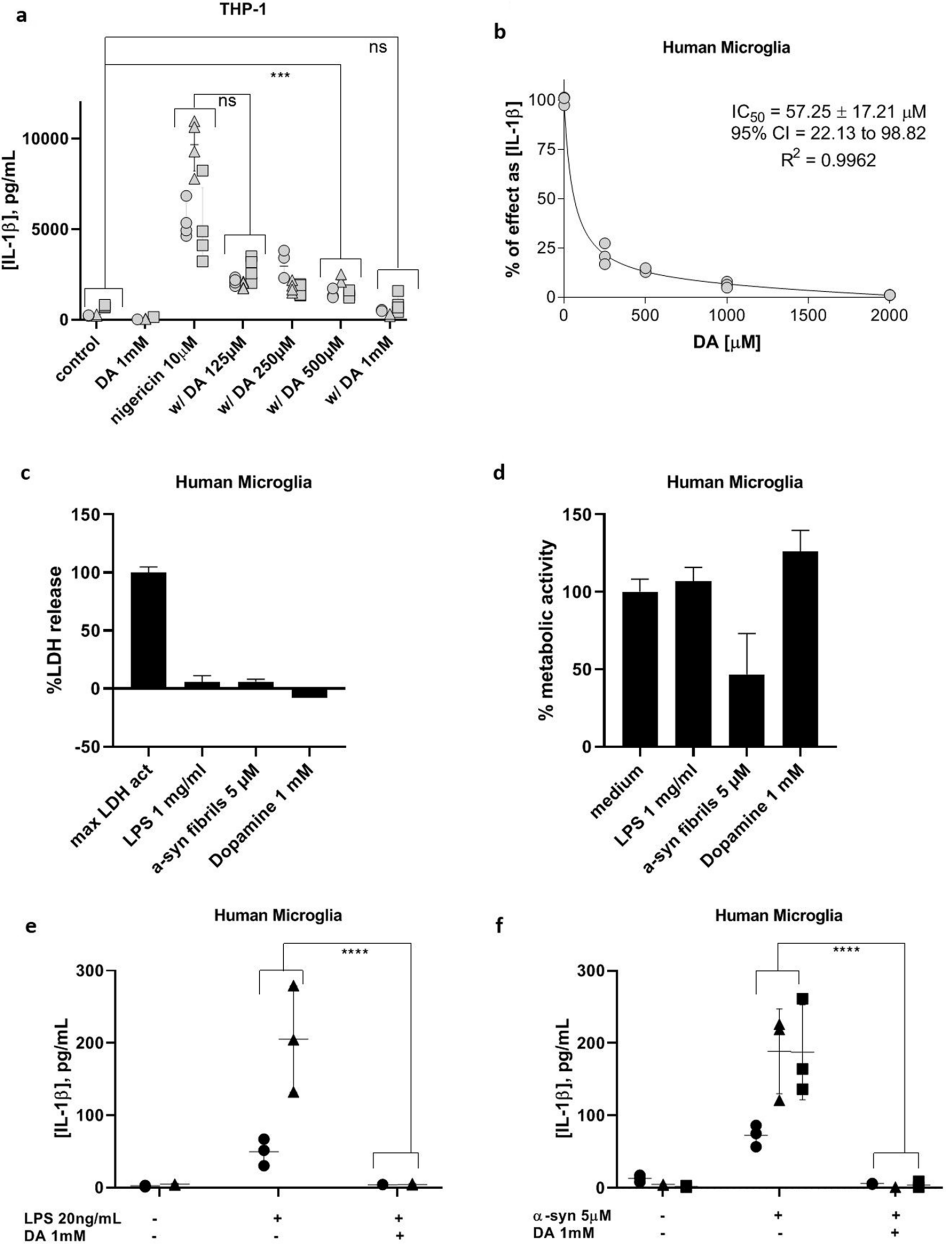
Dopamine inhibits IL-1β production by THP-1 cells and primary human microglia. **a** IL-1β output by PMA-differentiated THP-1 cells upon canonical inflammasome activation with nigericin (10 µM) in the presence or absence of a DA gradient (125 µM–1 mM; 3 experiments, *n* = 4). **b** Dose-inhibition curve indicating effect of DA on IL-1β output by primary human microglia upon canonical activation with LPS (20 ng/mL, 3.5 h) followed by nigericin (10 µM, 30 min.) in the presence or absence of a DA gradient (250 µM–2 mM, representative experiment). **c** The plasma membrane integrity of human microglia is not perturbed by DA at 1 mM overnight. Representative experiment. **d** Primary human microglia metabolic function is not affected by 1 mM DA overnight. Representative experiment. **e** DA inhibits non-canonical, LPS-mediated IL-1β production in primary human microglia (2 experiments, *n* = 3). **f** DA inhibits α-syn-mediated IL-1β production by primary human microglia (3 experiments, *n* = 3). ***p < 0.001, ns = not significant by two-way ANOVA with Bonferroni multiple testing correction. Shapes represent individual experiments

We have shown previously that primary human microglia are competent for short-term, canonical NLRP3 inflammasome activation by the established positive control stimuli LPS and nigericin [29]. To determine whether or not canonical inflammasome activation in human cells could be blocked by DA, both primary human microglia and the macrophage-like PMA-differentiated THP-1 cell model were activated with positive control stimuli in the presence of a DA gradient (Fig. 1). For THP-1 experiments, no separate LPS priming step was required, so DA was administered at the time of application of nigericin (30 min, Fig. 1a). For experiments with primary human microglia, DA was administered concomitantly with LPS (3.5 h) and these were left in the system upon the addition of nigericin (30 min, Fig. 1b). In both cell types and with both exposure times, DA exhibited a strong ability to inhibit canonical NLRP3 inflammasome activation as evidenced by progressive attenuation of IL-1β output. The only cytokine we examined, based on limited sample quantities, was IL-1β; thus, NF-κB-dependent mechanisms remain to be ruled out by assessing IL-6 or TNF-α secretion.

In primary human microglia, the apparent IC_50_ for DA-mediated inhibition of IL-1β secretion was calculated to be 57.25 ± 17.21 µM (Fig. 1b). We wanted to select a higher concentration than this for subsequent experiments to have an effect significantly higher than the intrinsic variability level due to the limited availability of the human samples. In the THP-1 experiments, the concentration of 1 mM DA, while significantly higher than physiological DA concentrations, was able to suppress IL-1β secretion from activated cells to a level not significantly different from baseline noise (Fig. 1a), so we chose to proceed with this concentration. No cytotoxicity was evident at this concentration of DA, even after overnight exposure (Fig. 1c, d).

We next investigated whether or not DA also affects non-canonical inflammasome activation and observed that similar inhibition of IL-1β secretion in the presence of DA was evident for longer term NLRP3 inflammasome activation induced by overnight treatment with LPS (Fig. 1e). Particularly relevant to PD, DA was also able to inhibit α-syn-mediated NLRP3 inflammasome activation (Fig. 1f).

### Dopamine-mediated inhibition of canonical NLRP3 inflammasome activation is not attributable to dopamine metabolites

**Fig. 2:**
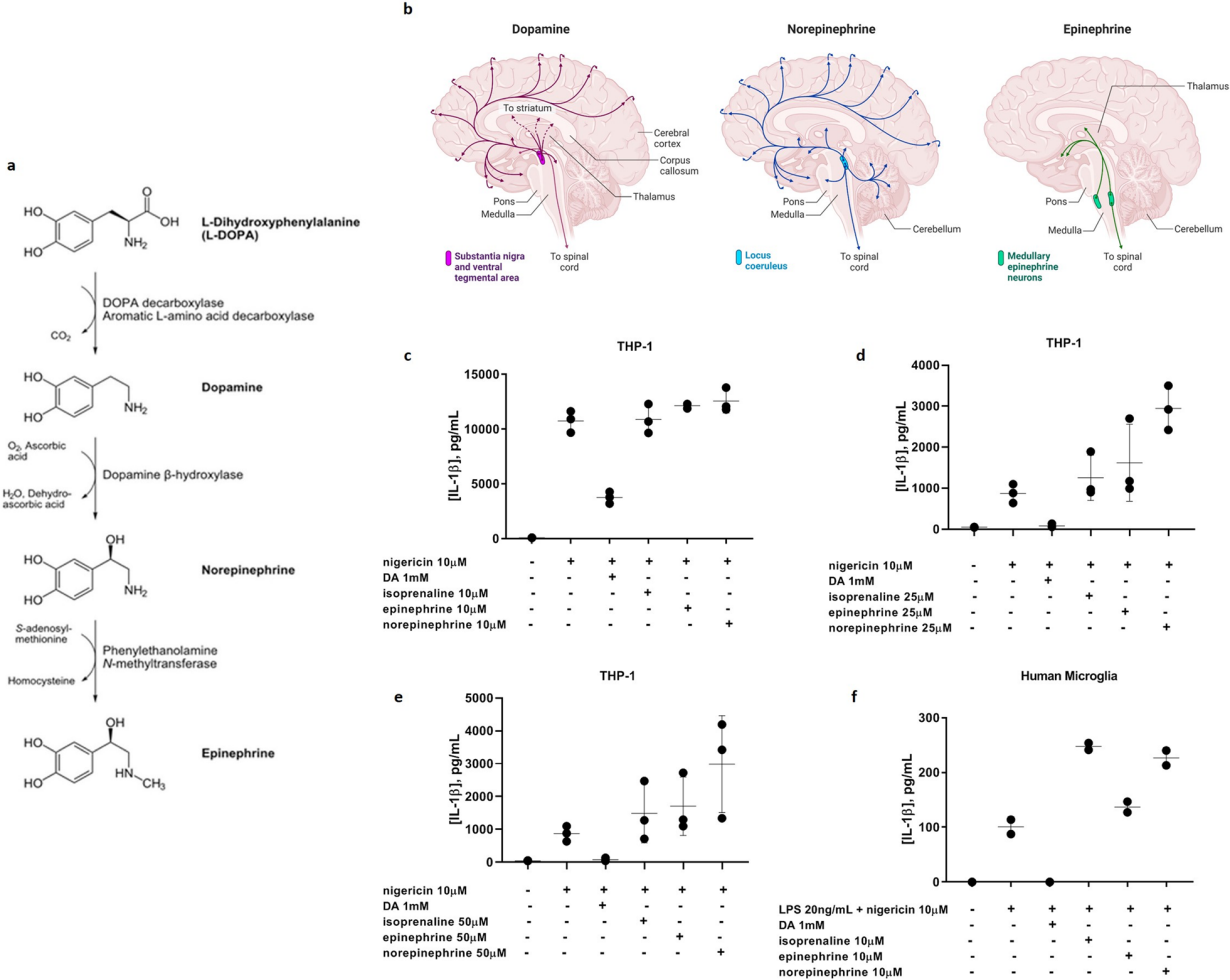
Lack of effect of catecholamine β-adrenoceptor ligands on inflammasome-induced IL-1β secretion from THP-1 cells and primary human microglia. **a** l-DOPA is catabolized sequentially to DA, norepinephrine, and epinephrine. Image “Biosynthesis of catecholamines” is licensed under CC BY 2.0. **b** Distribution patterns of DA, norepinephrine, and epinephrine in human brain. Image created in BioRender. **c**–**e** Canonical IL-1β production by THP-1 cells is inhibited with 1 mM DA but not by isoprenaline, epinephrine, or norepinephrine at 10 µM (**c**), 25 µM (**d**), or 50 µM (**e**). **f** Canonical IL-1β production by primary human microglia is also inhibited with 1 mM DA but not by isoprenaline, epinephrine, or norepinephrine at 10 µM. Representative experiments

DA, generated in the brain from tyrosine-derived or clinically administered l-DOPA, has a short extracellular half-life and is quickly metabolized further to norepinephrine and epinephrine (Fig. 2a). Norepinephrine in particular shows a similar distribution pattern in human brain to DA (Fig. 2b). To ascertain whether the inhibitory effect of DA on IL-1β secretion observed in Fig. 1 could be ascribed to norepinephrine or epinephrine rather than to DA itself, canonical NLRP3 inflammasome activation was performed on THP-1 cells and primary human microglia in the presence of DA, norepinephrine, epinephrine, or the β-adrenoceptor agonist isoprenaline (Fig. 2c–e). Neither the catecholamine byproducts nor the agonist showed any inhibitory effect on IL-1β secretion triggered by canonical NLRP3 inflammasome activation at any of the concentrations tested. While it cannot be completely ruled out that this was because the experimental concentrations of the three β-adrenoceptor binding compounds were limited relative to that of DA due to solubility issues, these observations suggest that the protective effect exhibited by DA is not the result of a downstream metabolite acting on β-adrenoceptors, but rather to the action of DA itself.

### l-DOPA inhibits canonical, non-canonical, and α-syn-induced NLRP3 inflammasome activation in primary microglia upon conversion to dopamine

**Fig. 3:**
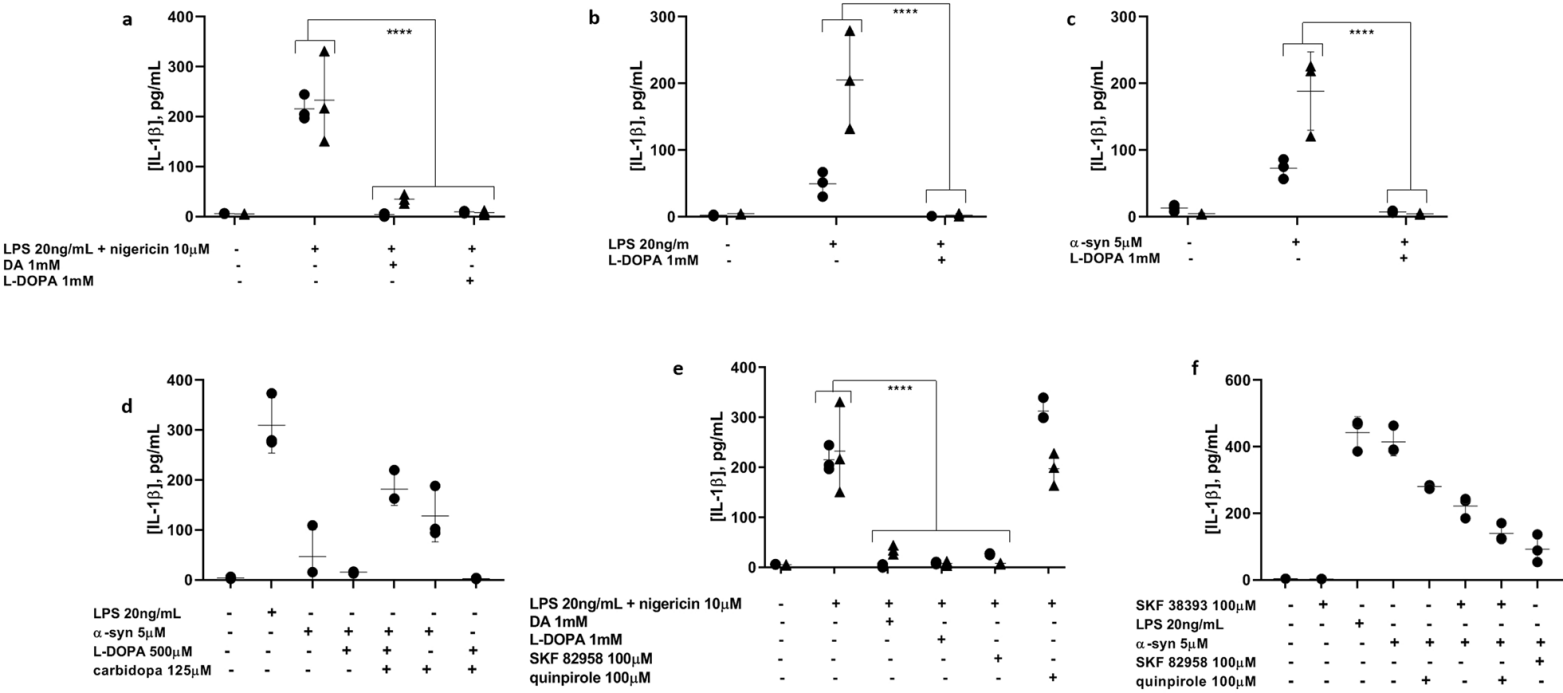
Co-administered l-DOPA is able to block IL-1β production by primary human microglia in response to canonical (**a**), non-canonical (**b**), and α-syn-mediated (**c**) NLRP3 inflammasome activation. 2 experiments, *n* = 3. **d** The protective effect of l-DOPA is reversed by the AADC inhibitor carbidopa, indicating that l-DOPA-mediated inhibition of IL-1β production is a result of the conversion of l-DOPA to DA. **e** DRD1 agonism with SKF82958 completely blocks IL-1β production by canonical NLRP3 inflammasome activation, similar to DA and l-DOPA, while DRD2 agonism with quinpirole has no effect. 2 experiments, *n* = 3. **f** DRD1 and DRD2 agonism both partially block IL-1β production induced by α-syn, suggesting a shared role for these receptors in α-syn-mediated NLRP3 inflammasome activation. Representative experiment. ****p < 0.0001 by two-way ANOVA with Bonferroni multiple testing correction. Shapes represent individual experiments

If DA is responsible for inflammasome inhibition, then its immediate upstream metabolic precursor, l-DOPA, also would be expected to show an inhibitory effect on IL-1β production. Indeed, l-DOPA demonstrated an ability similar to that of DA to block canonical NLRP3 inflammasome activation and IL-1β output in primary human microglia (Fig. 3a). l-DOPA was also able to inhibit non-canonical, LPS-mediated inflammasome activation (Fig. 3b) and α-syn-induced inflammasome activation (Fig. 3c) in primary human microglia. To determine whether this inhibition was due to the l-DOPA itself acting on the microglia or rather to l-DOPA-derived DA, the amino acid decarboxylase (AADC) inhibitor carbidopa was employed to block the decarboxylation of l-DOPA to DA. Upon inflammasome activation in the presence of both l-DOPA and carbidopa (in a 4:1 ratio), the protective effect of l-DOPA was reversed, indicating that downstream DA is responsible for preventing inflammasome activation (Fig. 3d).

### Dopamine binding to dopamine receptors signals for the inhibition of the NLRP3 inflammasome activation in primary human microglia

To rule out the possibility that the observed inhibitory effect of DA on inflammasome-mediated IL-1β output was due to binding or solvating of the activating stimuli by DA or l-DOPA rather than to DA signaling, and to resolve more clearly the mechanism by which DA acts on primary human microglia to block IL-1β production, the DA receptor agonists SKF82958, SKF38393, and quinpirole were utilized. For canonical activation, SKF82958 distinctly mirrored the IL-1β blocking effects of DA and l-DOPA. SKF38393 had a similar, although less significant, effect, whereas quinpirole had no effect (Fig. 3e). This pattern suggests that DRD1, and not DRD2, is the receptor through which DA acts to inhibit short-term, canonical NLRP3 inflammasome activation. However, for longer term, α-syn-mediated inflammasome activation, coincubation with either SKF82958 or quinpirole resulted in a partial inhibitory effect on IL-1β production (Fig. 3f), which supports a shared role for DRD1 and DRD2 signaling in the DA-mediated inhibition of α-syn-induced NLRP3 inflammasome activation in primary human microglia.

### High extracellular K^+^ blocks canonical, non-canonical, and α-syn-mediated NLRP3 inflammasome activation in primary human microglia

**Fig. 4:**
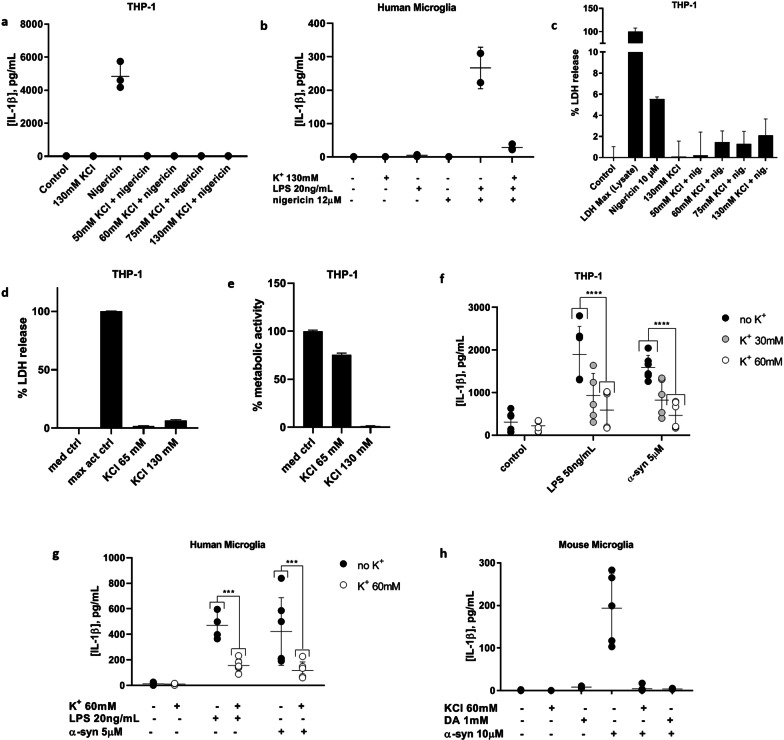
Elevated extracellular K^+^ inhibits NLRP3 inflammasome activation in THP-1 cells and primary human and mouse microglia. **a** Canonical inflammasome activation is blocked in THP-1 cells with a range of extracellular K^+^ concentrations (130–50 mM). Representative experiment. **b** 130 mM extracellular K^+^ blocks canonical inflammasome activation in primary human microglia. Representative experiment. **c** 130 mM extracellular K^+^ is non-toxic to THP-1 cells upon short-term (4 h) exposure. Representative experiment. **d**, **e** Overnight exposure of THP-1 cells to 130 mM extracellular K^+^ does not affect plasma membrane integrity (**d**) but does affect metabolic function (**e**), suggesting cytotoxicity at this timepoint, whereas 65 mM K^+^ has little effect by either measure. Representative experiments. **f** Both LPS- and α-syn-mediated IL-1β production by THP-1 cells are inhibited by 30 or 60 mM extracellular K^+^. 2 experiments, *n* = 3. **g** Both LPS-and α-syn-mediated IL-1β production by primary human microglia are also blocked by 60 mM extracellular K^+^. 2 experiments, *n* = 3. **h** Both 1 mM DA and 60 mM extracellular K^+^ inhibited IL-1β production induced by overnight treatment of primary mouse microglia with 10 µM α-syn. ***p < 0.001 by two-way ANOVA with Bonferroni multiple testing correction

An extracellular K^+^ concentration of 130 mM, approximating intracellular levels, worked well to block IL-1β production in canonical inflammasome activation experiments with an exposure time of only 4 h in total in THP-1 cells (Fig. 4a) and primary human microglia (Fig. 4b). Unlike DA, however, these higher K^+^ concentrations resulted in some cytotoxicity for the non-canonical LPS and α-syn activation experiments with THP-1 cells upon longer term (overnight) exposures (Fig. 4c–e). A maximum K^+^ concentration of 65 mM was determined to be non-toxic, so 60 mM was used for the longer term experiments with LPS and α-syn. In primary human microglia and THP-1 cells, 60 mM KCl added to the culture medium was largely able to block non-canonical and α-syn-mediated inflammasome activation (Fig. 4f, g).

### α-Syn-mediated NLRP3 inflammasome activation is also blocked by both DA and elevated extracellular K^+^ in primary mouse microglia

While it has been demonstrated previously that canonical NLRP3 inflammasome activation can be blocked in mouse microglia by DA [32] and in macrophages by elevated extracellular K^+^ [44], the effects of these inhibitors have been reported for neither primary human microglia nor non-canonical inflammasome activation, including that mediated by α-syn, in any species to our knowledge. As expected, both 60 mM KCl and 1 mM DA were able to prevent IL-1β production from primary mouse microglia (Fig. 4h), consistent with primary human microglia.

### SYN120 tg mice show increased microglial NLRP3 inflammasome component protein immunopositivity that is inhibited by DRD1 agonism

At 12 months of age, SYN120 tg mice display marked accumulation of insoluble α-syn aggregates in the nigrostriatal system and significant failure of basal and depolarization-dependent putaminal dopamine release as well as microglia activation [37, 38]. Given the relationship between α-syn, NLRP3 inflammasome activation, and DA-mediated inhibition that we observed in primary human microglia, we assessed whether the SYN120 tg mice, which were previously described to exhibit microglial activation in the *substantia nigra* at 12 months of age [37], could show signs of increased inflammasome activation in this time frame in comparison to wild-type (WT) mice.

**Fig. 5:**
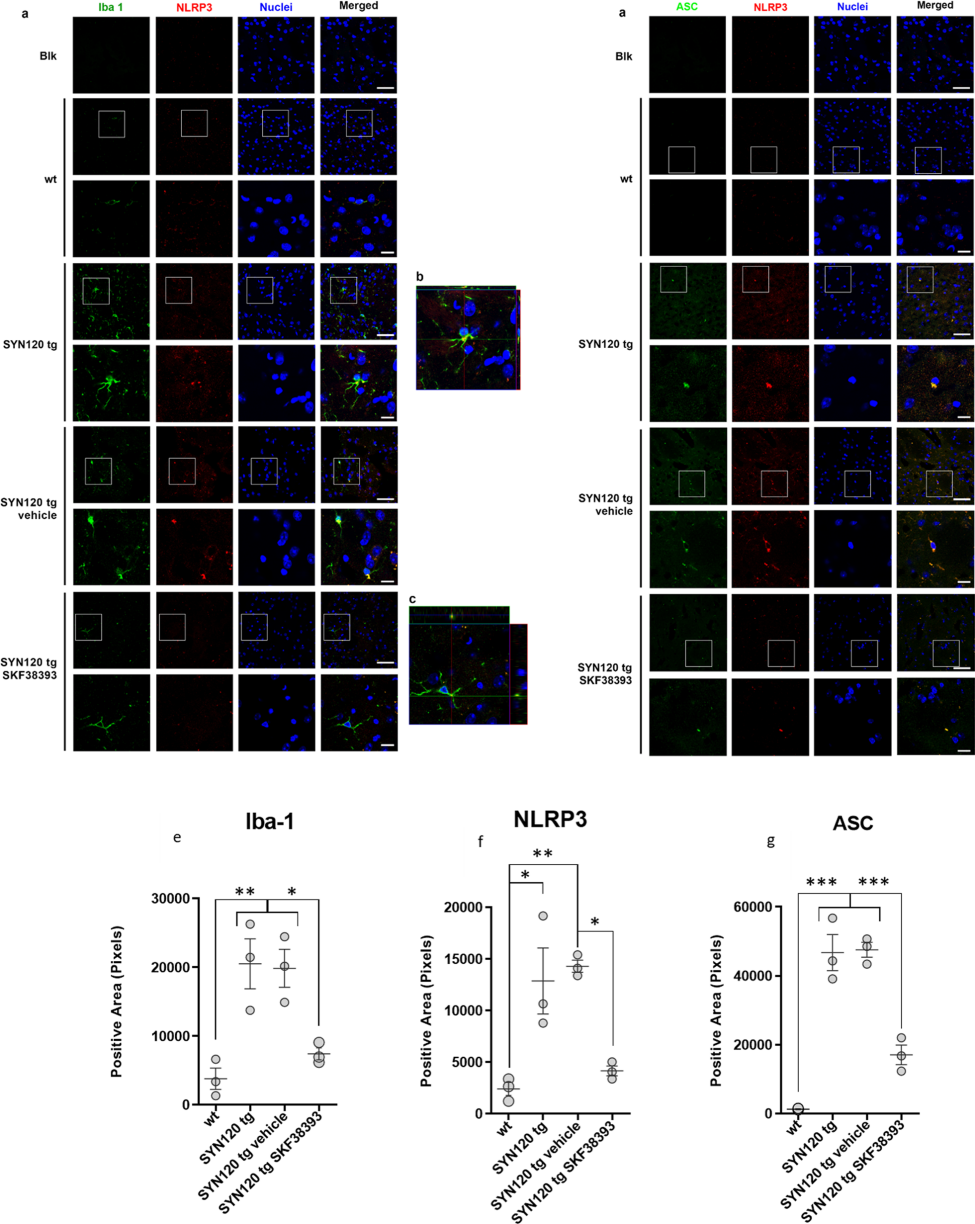
Strong microglia activation and increased microglial inflammasome activation evident in SYN120 tg mice as compared to WT mice is counteracted by DRD1 agonism. **a** Representative confocal images showing Iba-1 (green) and NLRP3 (red) immunopositivity in striatum of 12-month-old WT mice, SYN120 tg mice, SYN120 tg mice treated for 1 month with saline vehicle (SYN120 tg vehicle), and SYN120 tg mice treated for 1 month with 10 mg/kg/day SKF38393 (SYN120 tg SKF38393). Scale bar 50 µm. **b** Z-stack reconstruction of ~ 10 µm showing that NLRP3 signal is present in Iba-1-positive cells (colocalization in yellow). **c** Z-stack reconstruction of ~ 10 µm showing decreased NLRP3 signal in Iba-1-positive cells in striatum of 12-month-old SYN120 mice after 1 month of SKF38393 treatment. **d** Representative images showing NLRP3 (red) and ASC (green) immunopositivity in striatum of 12-month-old WT mice, SYN120 tg mice, SYN120 tg vehicle mice, and SYN120 tg SKF38393 mice. The overlap in NLRP3 and ASC immunopositivity (yellow) is suggestive of inflammasome activation. Scale bar 50 µm. **e**–**g** Graphs represent the quantification of the indicated protein [Iba-1 (**e**), NLRP3 (**f**), or ASC (**g**)] in striatum slices of 12-month-old WT, SYN120 tg, SYN120 tg vehicle, and SYN120 tg SKF38393 mice based on immunopositive area in field to confirm observations from confocal images in **a**–**d**. *p < 0.05 by one-way ANOVA, *n* = 3 animals for each group

To this purpose, double immunohistochemistry experiments were performed to label NLRP3 in cells positive for the microglia/macrophage marker Iba-1 and to estimate ASC/NLRP3 co-localization in striatal slices from 12-month-old WT and Tg120 mice. In addition, 11-month-old SYN120 tg mice were treated with the blood–brain barrier-permeable DRD1 partial agonist SKF38393 to evaluate in vivo the DA-dependent modulation of inflammasome activation. The choice to use SKF38393 rather than SKF82958 for in vivo treatments was governed by evidence showing that this latter also showed D2 agonist activity when administered in rats [52]. We found that SYN120 tg mice exhibited activated microglia which presented with NLRP3 immunoreactivity (Fig. 5a) as confirmed by the orthogonal projection of the z-stack acquisition (Fig. 5b). Moreover, we found that NLRP3-positive signal observed in SYN120 tg mice co-localized with ASC immunoreactivity, as seen in the orthogonal projections in Fig. 5d. Taken together, this evidence is indicative of inflammasome activation within microglial cells in the brains of SYN120 tg mice, but not WT mice, at 12 months of age. Consistently, from the quantitative analysis of Iba-1 (Fig. 5e), NLRP3 (Fig. 5f), and ASC immunopositivity (Fig. 5g), we found that these proteins were significantly increased in the striatum of SYN120 tg mice when compared to WT littermates. Interestingly, 1 month of treatment with the DRD1 agonist SKF38393 was able to hamper the activation of the microglia in SYN120 tg mice, as shown in Fig. 5a–d. The stimulation of DRD1 induced a significant decrease of NLRP3, ASC, and Iba-1 immunopositive signal (Fig. 5e–g). However, whether IL-1β levels are also increased in SYN120 tg mice remains to be explored.

## Discussion

The findings of this study provide evidence for the modulatory function of DA on NLRP3 inflammasome activation, which is known to link major aspects of PD pathology (neuroinflammation involving activated microglia, α-syn pathology, and dopaminergic neurodegeneration), in primary human microglia as well as the THP-1 model. Moreover, we show that in the SYN120 tg mouse model of PD at a pathological stage exhibiting striatal dopaminergic failure, the re-establishment of D1 receptor activation through 1-month i.p. daily treatment with SKF38393 is able to reduce NLRP3 inflammasome activation. The data suggest (i) that DA blocks canonical, non-canonical, and α-syn-induced microglial NLRP3 inflammasome activation in primary human microglia; (ii) that through its decarboxylation to DA, l-DOPA is able to recapitulate this inhibition, whereas norepinephrine and epinephrine downstream of DA are not; (iii) that DA exerts its inhibitory effect through DRD1 signaling for canonical inflammasome activation and a mixture of DRD1 and DRD2 signaling for non-canonical and α-syn-mediated activation; and (iv) that elevated extracellular K^+^ is also able to block all three modes of inflammasome activation.

DA has been shown to block canonical NLRP3 inflammasome activation and IL-1β secretion induced by various stimuli in mouse microglia and in the MPTP parkinsonism model in vivo [32], though α-syn-mediated inflammasome activation was not tested. The present study illustrates the ability of DA to block not only canonical but also non-canonical and α-syn-mediated NLRP3 inflammasome activation in primary human microglia, thus serving as an endogenous, PD-relevant regulator of microglial inflammasome activation. l-DOPA, upstream of DA in the catecholamine synthesis pathway, displayed a similar ability to inhibit IL-1β secretion from primary human microglia that was reversible upon blocking the conversion of l-DOPA to DA. On the other hand, norepinephrine and epinephrine, downstream of DA, were unable to do so. The present study did not address other downstream metabolites of DA, such as 3,4-dihydroxyphenylacetic acid (DOPAC) and homovanillic acid (HVA), so the effects of these cannot yet be ruled out. These observations suggest that DA itself is the modulator of NLRP3 inflammasome activation in these experiments rather than its downstream metabolites.

In these experiments, we utilized concentrations of dopamine which were higher than physiological levels. Our initial decision to proceed with the chosen dopamine concentrations were based in part on the argument presented by [32], who also used higher-than-physiological concentrations of dopamine in their inflammasome inhibition experiments. The authors justified this by performing two different treatment protocols on primary mouse bone marrow-derived macrophages, both experiments with the same total dopamine concentration: one with a single high dose of dopamine (45 µM or 90 µM) as compared to one with repeated low doses of dopamine (1.5 µM or 3 µM, 30 times over 5 min). The single high-dose dopamine treatment could not inhibit canonical (nigericin-induced) NLRP3 inflammasome activation at 45 µM or 90 µM dopamine, but higher concentrations of 250 µM dopamine could and were thus utilized for their subsequent single-dose experiments. On the other hand, the repeated low-dose protocol successfully inhibited inflammasome activation, as did a single low-dose treatment in the presence of monoamine oxidase (MAO) and catecholamine *O*-methyltransferase (COMT) inhibitors, and this led the authors to suggest that the dopamine was indeed inhibitory, and that the requirement for the high single dose was the result of the short lifetime of the dopamine leading to its rapid oxidation after its application to the cells. Previous research [53] presented an in vitro analysis of the lifetime of dopamine and various oxidation products thereof. In a controlled environment, the time scale for dopamine oxidation was anywhere between 30 and 120 min, and we reason that in the presence of cells the reaction would be faster still.

Release of DA for diffusion-based volume transmission signaling from dopaminergic neurons in the midbrain occurs somatodendritically in the substantia nigra (SN) and the ventral tegmental area (VTA) as well as axonally in the striatum [54–62]. Somatodendritically, DA release takes place in a highly localized action potential- and K^+^ channel-dependent manner [58, 59]. DA acts on the DA receptors of surrounding cells to exert its effects, so the present study explored with the help of agonists the participation of DA receptors in the NLRP3 inflammasome inhibition process in primary human microglia. The previously demonstrated ameliorative effect in mouse microglia of DA on canonical inflammasome activation was attributed to DA binding to DRD1 and signaling downstream for the ubiquitination of the NLRP3 protein via the E3 ligase MARCH7, leading to the degradation of NLRP3 by autophagy and thus control of NLRP3 protein levels [32]. Our observations that DA can inhibit various modes of inflammasome activation including canonical, non-canonical, and α-syn-mediated, is consistent with this, as NLRP3 is involved with all three modes of activation. DA is a potent chemotactic signal for activated primary adult human microglia, which have been shown to express DA receptors DRD1, DRD2, DRD3, and DRD4 with the relative gene expression levels DRD2 > DRD4 > DRD1 ≈ DRD3 [36]. For these reasons, DRD1 and DRD2 were considered the most likely candidates for agonist experiments, so we performed experiments with SKF 82958, a full DRD1 agonist [63], SKF38393, a partial DRD1 agonist [51, 52], and quinpirole, a selective DRD2 agonist [64]. Under canonical NLRP3 inflammasome activation conditions in primary human microglia, DRD1 appeared to be the sole target receptor for DA, whereas for α-syn-mediated activation, signaling via DRD1 and DRD2 both appeared to contribute to the inhibitory effect of DA. However, because we did not specifically characterize the gene expression of DA receptors on our microglia, it is possible that other DA receptors may be involved in this process. Indeed, human microglia are known to undergo expression profile changes post-isolation [65], rendering them an imperfect representation of in situ microglia.

Elevated extracellular K^+^ blocked canonical, non-canonical, and α-syn-mediated NLRP3 inflammasome activation in the present study, presumably by disrupting the ion gradient that normally allows for K^+^ efflux from the cell in a similar manner to that described for mouse macrophages [44]. To our knowledge, this has not been shown previously for primary human or mouse microglia. K^+^ efflux upon microglial NLRP3 inflammasome activation has been suggested to proceed via the voltage-gated K^+^ channel Kv1.3, as specifically blocking this channel prevented LPS-induced, inflammasome-mediated IL-1β secretion from primary rat microglia [66]. Whether K^+^ efflux from primary human microglia upon inflammasome activation also proceeds through Kv1.3 remains to be seen. DA release in striatal axon terminals has been shown to be controlled by voltage-gated Kv1 channels including Kv1.3 [47] and in the SN by other voltage-gated K^+^ channels [58], linking K^+^ flux to DA release, but any potential effect of microglial Kv1.3 on dopaminergic neuronal Kv1.3 function has not been explored. However, microglial Kv1.3 activity has been documented to have a more general effect on neurons in terms of toxicity, where LPS-activated microglia kill neurons through a reactive oxygen species-mediated mechanism that is inhibited by Kv1.3 channel blockers [67, 68]. Thus, K^+^ efflux from microglia undergoing NLRP3 inflammasome activation may contribute to the release of DA from neighboring neurons, possibly through the action of Kv1.3.

**Fig. 6:**
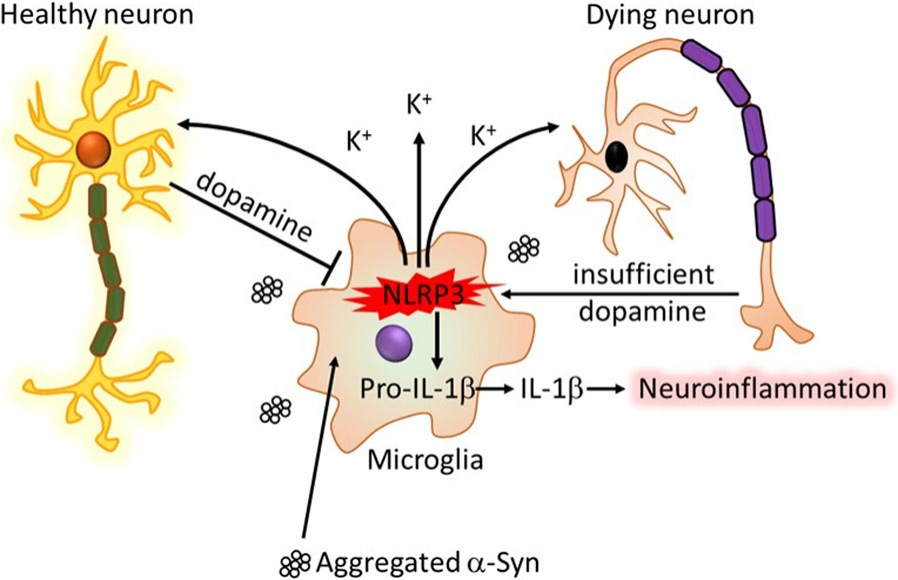
Schematic diagram of dopamine as a countersignal to α-syn-mediated NLRP3 inflammasome activation in PD. Fibrillar α-syn is taken up by microglia, leading to K^+^ efflux-dependent activation of the NLRP3 inflammasome. The efflux of K^+^ could affect the Kv1.3-mediated or toxicity-induced release of DA from proximal dopaminergic neurons. DA can then bind to microglial DRDs to curb activation of the inflammasome. Sufficient DA in the system would keep this interaction under control, but if DA is depleted over time by the microglia, the imbalance could allow for the progression of neurotoxicity and inflammation

We suggest the following framework, illustrated schematically in Fig. 6: upon escalating inflammasome activation with resultant IL-1β secretion by the microglia population in response to α-syn aggregates or other stimuli, K^+^ efflux from microglia in the immediate environment may locally affect DA release from adjacent dopaminergic neurons, possibly via voltage-gated Kv1.3 channels on both cell types. DA released somatodendritically from neurons in response to this trigger, or non-specifically as a result of microglia-mediated breakdown of the neurons, could then bind to microglial DA receptors as a negative feedback signal for inflammasome activation, siphoning DA from finite neuronal stores. As long as sufficient DA is available, functional signaling by DA and its metabolites could be maintained in the midbrain, but as the neuronal DA supply is gradually exhausted by the microglia, this would be progressively impaired. At the same time, as DA availability diminishes, the inflammasome activation rate could surpass that of DA-mediated inhibition to enhance pathology, an imbalance that could be further exacerbated by the potent chemotactic ability of extracellular DA to recruit additional activated microglia as demonstrated by [36].

## Conclusions

The results of the present study demonstrate the ability of DA, by signaling through its receptors, to inhibit K^+^ efflux-dependent, NLRP3 inflammasome-mediated IL-1β secretion induced by α-syn in primary human and mouse microglia. They also demonstrate that SYN120 tg mice, which present with insoluble α-syn aggregation pathology, microglial activation, and failure of DA release, display evidence of increased microglial NLRP3 inflammasome activation in comparison to WT counterparts, and that DRD1 signaling abates this inflammasome activation. Taken together, these results indicate that DA could serve as an endogenous inhibitor for the NLRP3 inflammasome activation that is associated with multiple facets of PD, including α-syn pathology, neuroinflammation, and dopaminergic neurodegeneration.

Overall, the interactions proposed in the mechanistic hypothesis in Fig. 6 could explain the key role of microglial NLRP3 inflammasome activation in PD-related α-syn pathology, neuroinflammation, and dopaminergic neurodegeneration described by others [12, 69], and offer a potential mechanistic basis for the particular vulnerability of SN dopaminergic neurons in PD. Moreover, the data presented in this study support the ongoing development of NLRP3 inhibitors or other inflammasome inhibitors for clinical application to curtail or slow down progression of PD pathology. Such therapeutic measures in combination with the standard l-DOPA administration that increases the availability of DA in the SN and striatum may serve as a disease-modifying treatment strategy for PD.

## Data Availability

The data that support the findings of this study are available from the corresponding author upon reasonable request.

## Abbreviations

PD: Parkinson’s disease
α-syn: α-Synuclein
IL-1β: Interleukin-1β
NLRP3: NACHT domain-, leucine-rich repeat (LRR)-, and pyrin domain-containing protein 3
MPTP: 1-Methyl-4-phenyl-1,2,3,6-tetrahydropyridine
l-DOPA: Levodopa
DRD1 and DRD2: Dopamine receptor D1 and dopamine receptor D2
WT: Wild type
DA: Dopamine
LRR: Leucine-rich repeat
CARD: Caspase activation and recruitment domain
ASC: Apoptosis-associated speck-like protein containing a CARD
Caspase-1: Cysteine-aspartate protease-1
PMA: Phorbol-12-myristate-13-acetate
LPS: Bacterial lipopolysaccharide
ELISA: Enzyme-linked immunosorbent assay
LDH: Lactate dehydrogenase leakage assay
MTT: Methyl tetrazolium assay
ANOVA: Analysis of variance
*K*_app_: Apparent constant
AADC: Amino acid decarboxylase
KCl: Potassium chloride
tg: Transgenic
SN: Substantia nigra
VTA: Ventral tegmental area
DOPAC: 3,4-Dihydroxyphenylacetic acid
HVA: Homovanillic acid
MAO: Monoamine oxidase
COMT: Catecholamine *O*-methyltransferase
